# Supplementary material for: In vivo assessment of the neural substrate linked with vocal imitation accuracy
Source: eLife. 2020 Mar 20;9:e49941. doi: 10.7554/eLife.49941 (PMC7083600; doi:10.7554/eLife.49941)
Supplement: Supplementary file 1. — FA stands for Fractional Anisotropy, one of the DTI metrics. The statistical maps were assessed at pFWE <0.05 and kE ≥5 voxels. ‘Interaction age*sex’ was assessed in a flexible factorial design and serves to unveil brain regions that display a sex-dependent trajectory during ontogeny ‘Time effect’ was assessed for each sex separately in a flexible factorial design and serves to unveil brain regions that change microstructurally over time. The ‘Cluster level’ and ‘Peak level’ columns refer to respectively the p-value (after ‘Family Wise Error’ correction for multiple comparisons) of the clusters and cluster extent (kE), and p- and F-values of the peak voxel of the clusters respectively, provided by SPM (see material and methods section). [file elife-49941-supp1.docx]

**Supplementary file 1: Clusters displaying a time effect for FA in male and female zebra finch brains.**

| Statistical test | Cluster-ROI | | Cluster level | | Peak level | |
| --- | --- | --- | --- | --- | --- | --- |
|  |  |  | p_FWE_ | k_E_ | p_FWE_ | F |
| Interaction age * sex | Arcopallium | Left | <0.001 | 21 | <0.001 | 16.49 |
|  |  | Right | <0.001 | 28 | <0.001 | 25.39 |
|  | Rostro-lateral Area X surroundings | Left | <0.001 | 34 | <0.001 | 10.33 |
|  |  | Right | 0.001 | 20 | 0.002 | 8.76 |
|  | Caudal area X surroundings | Left | <0.001 | 11 | <0.001 | 9.62 |
|  |  | Right | <0.001 | 16 | 0.001 | 9.08 |
| Time effect males | tOM | Left | <0.001 | 17970 | <0.001 | 103.11 |
|  |  | Right |  |  | <0.001 | 63.71 |
|  | LaM | Left |  |  | <0.001 | 87.26 |
|  |  | Right |  |  | <0.001 | 65.15 |
|  | Arcopallium | Left |  |  | <0.001 | 46.01 |
|  |  | Right |  |  | <0.001 | 68.99 |
|  | LFS | Left |  |  | <0.001 | 54.45 |
|  |  | Right |  |  | <0.001 | 57.92 |
|  | tFA | Left |  |  | <0.001 | 49.48 |
| Time effect females | tOM | Left | <0.001 | 11979 | <0.001 | 110.44 |
|  |  | Right |  |  | <0.001 | 69.67 |
|  | tFA | Left |  |  | <0.001 | 51.09 |
|  | FPL | Left |  |  | <0.001 | 56.90 |
|  |  | Right |  |  | <0.001 | 49.69 |
|  | LaM | Left |  |  | <0.001 | 71.91 |
|  |  | Right | <0.001 | 567 | <0.001 | 51.52 |
|  | LFS | Left | <0.001 | 126 | <0.001 | 26.80 |
