# Supplementary material for: In vivo assessment of the neural substrate linked with vocal imitation accuracy
Source: eLife. 2020 Mar 20;9:e49941. doi: 10.7554/eLife.49941 (PMC7083600; doi:10.7554/eLife.49941)
Supplement: Supplementary file 2. — To test for a main effect of age, a mixed-effect model (n = 14–16; details in Methods section) was set up for each song score separately with age as fixed effect, subject as random effect, subject*age as random slope and –only for statistical analyses on the syllable feature level– syllable identity as random effect nested within subject. To test for a main effect of tutor, a mixed-effect model was executed for song similarity or sequence stereotypy separately with tutor as fixed effect and subject as random effect. The restricted Maximum Likelihood method was used to fit the data and significance was assessed using F-tests with the Kenward-Roger approximation. [file elife-49941-supp2.docx]

**Supplementary file 2: Summary of the mixed-effect model analyses on the song scores.**

| **Syllable features (Main effect of age)** | |
| --- | --- |
| inter-syllable interval duration | *p*<0.0001 *F_(3,38.2)_*=13.8789 |
| syllable duration | *p*=0.5534 *F_(3,37.7)_*=0.7078 |
| Wiener entropy | *p*=0.0032 *F_(3,37.8)_*=5.4803 |
| pitch | *p*=0.8897 *F_(3,37.7)_*=0.2088 |
| mean frequency | *p*=0.1289 *F_(3,38.6)_*=2.0078 |
| peak frequency | *p*=0.0649 *F_(3,38.6)_*=2.6144 |
| goodness of pitch | *p*=0.2211 *F_(3,38.1)_*=1.5348 |
| FM | *p*=0.3279 *F_(3,37.4)_*=1.1867 |
| AM | *p*=0.5470 *F_(3,37.4)_*=0.7189 |
| **Standard deviation of syllable features (Main effect of age)** | |
| inter-syllable interval duration | *p*<0.0001 *F_(3,25.4)_*= 20.8043 |
| syllable duration | *p*=0.0064 *F_(3,34.3)_*=4.8560 |
| Wiener entropy | *p*=0.0021 *F_(3,36.9)_*=5.9192 |
| pitch | *p*=0.5824 *F_(3,41.4)_*=0.6583 |
| mean frequency | *p*=0.0132 *F_(3,37.5)_*=4.0891 |
| peak frequency | *p*=0.0280 *F_(3,38.1)_*=3.3787 |
| goodness of pitch | *p*=0.0621 *F_(3,33.1)_*=2.6910 |
| FM | *p*=0.0700 *F_(3,33.2)_*=2.5803 |
| AM | *p*=0.0982 *F_(3,41.5)_*=2.2367 |
| **Motif features (Main effect age)** | |
| song sequence stereotypy | *p*=0.0052 *F_(3,38.4)_*=4.7904 |
| % similarity | *p*=0.0251 *F_(3,37.0)_*=3.4890 |
| **Motif features (Main effect tutor)** | |
| song sequence stereotypy | *p*=0.1389 *F_(7,7.3)_*=2.3350 |
| % similarity | *p*=0.0159 *F_(7,6.1)_*=6.7597 |
