# Supplementary material for: In vivo assessment of the neural substrate linked with vocal imitation accuracy
Source: eLife. 2020 Mar 20;9:e49941. doi: 10.7554/eLife.49941 (PMC7083600; doi:10.7554/eLife.49941)
Supplement: Supplementary file 3. — ‘>65–68%’ indicates that the birds always sung song copies with a song similarity to tutor song score of at least 68%, while ‘<65–68%’ indicates that birds always sung songs with song similarity score lower than 65–68% similarity to tutor song. The 65–68% threshold was chosen arbitrarily based on the overall performance of the birds within the study. [file elife-49941-supp3.docx]

**Supplementary file 3: Number of syllables in the songs of good and bad learners.**

| **> 65-68% similarity** | | **< 65-68% similarity** | |
| --- | --- | --- | --- |
| Bird ID | Motif structure | Bird ID | Motif structure |
| 03 | ABCD | 05 | ABCD |
| 17 | ABCDEF | 07 | ABCDE |
| 18 | ABCDEF | 06 | ABC |
| 20 | ABCD | 08 | ABCDE |
| 21 | ABC | 09 | ABCDE |
| 23 | ABCD | 10 | ABCDE |
| 31 | ABC | 35 | ABC |
