# Supplementary material for: In vivo assessment of the neural substrate linked with vocal imitation accuracy
Source: eLife. 2020 Mar 20;9:e49941. doi: 10.7554/eLife.49941 (PMC7083600; doi:10.7554/eLife.49941)
Supplement: Supplementary file 4. — FA stands for Fractional Anisotropy, one of the DTI metrics. This table summarises the outcome of the voxel-based multiple regression based on 54 data points (12 birds with 4 time points and 2 birds with 3 time points). The ‘Cluster’ and ‘Peak’ columns refer to two different levels of assessing significance, respectively cluster-based inference and peak- or single voxel-based inference where the T- and p-value of the voxel with highest significance of the cluster is reported. Only clusters surviving pFWE <0.05 and kE > 5 voxels were considered significant. Grey font refers to clusters that were only visible when exploring the data at an exploratory statistical threshold, that is p uncorrected <0.001 and kE > 40 voxels. At this lower statistical threshold for visual assessment of the statistical maps, only clusters that still appeared significant with FWE correction at the cluster and peak level were considered relevant. The cluster extent for the clusters in the right tFA and right NCM are not reported as they could only be observed with an exploratory threshold (which influences cluster extent). [file elife-49941-supp4.docx]

**Supplementary file 4: Summary of the voxel-based multiple regressions (% similarity and FA).**

| **Correlation between** | **Cluster** | **Hemisphere** | **Cluster** | | **Peak** | |
| --- | --- | --- | --- | --- | --- | --- |
|  |  |  | **k_E_** | ***p_FWE_*** | ***T*** | ***p_FWE_*** |
| **% similarity and FA** | **tFA** | Left | 13 | 0.002 | 6.81 | <0.001 |
|  |  | Right | / | 0.034 | 6.42 | 0.001 |
|  | **NCM** | Left | 5 | 0.001 | 5.69 | 0.019 |
|  |  | Right | / | 0.024 | 5.55 | 0.032 |
|  | **VP** |  | 479 | <0.001 | 6.38 | 0.002 |
