# Supplementary material for: In vivo assessment of the neural substrate linked with vocal imitation accuracy
Source: eLife. 2020 Mar 20;9:e49941. doi: 10.7554/eLife.49941 (PMC7083600; doi:10.7554/eLife.49941)
Supplement: Supplementary file 5. — ‘log mwj’ refers to the log-transformed, modulated and warped jacobian determinants. This table summarises the outcome of the voxel-based multiple regression based on 54 data points (12 birds with 4 time points and 2 birds with 3 time points). The ‘Cluster’ and ‘Peak’ columns refer to two different levels of assessing significance, respectively cluster-based inference and peak- or single voxel-based inference where the T- and p-value of the voxel with highest significance of the cluster is reported. Only clusters surviving pFWE <0.05 and kE > 5 voxels were considered significant. [file elife-49941-supp5.docx]

**Supplementary file 5: Summary of the voxel-based multiple regressions (% similarity and log mwj).**

| **Correlation between** | **Cluster** | **Hemisphere** | **Cluster** | | **Peak** | |
| --- | --- | --- | --- | --- | --- | --- |
|  |  |  | **k_E_** | ***p_FWE_*** | ***T*** | ***p_FWE_*** |
| **% similarity and log mwj** | **VP** |  | 2022 | <0.001 | 8.06 | <0.001 |
|  | **CM** | Left | 1701 | <0.001 | 7.10 | 0.001 |
|  |  | Right | 3465 | <0.001 | 7.42 | <0.001 |
