# Supplementary material for: In vivo assessment of the neural substrate linked with vocal imitation accuracy
Source: eLife. 2020 Mar 20;9:e49941. doi: 10.7554/eLife.49941 (PMC7083600; doi:10.7554/eLife.49941)
Supplement: Supplementary file 6. — 'log mwj’ refers to the log-transformed, modulated and warped jacobian determinants; FA stands for Fractional Anisotropy, one of the DTI metrics. rmcorr’ is the repeated-measures correlation analysis. FDR rate = 0.05; number of tests = 8; i is the rank, m is the total number of tests and Q is the false discovery rate set at 0.05. Only those tests that survive FDR correction for multiple comparisons are highlighted in bold. ‘ [file elife-49941-supp6.docx]

**Supplementary file 6: Benjamini-Hochberg FDR correction for multiple comparisons of rmcorr analyses.**

| **MRI parameter** | **Cluster-based ROI** | **Hemisphere** | ***p* value** | **rank** | **(*i*/*m*)*Q*** |
| --- | --- | --- | --- | --- | --- |
| FA | VP |  | **0.0010** | **1** | **0.0063** |
| Log mwj | VP |  | **0.0057** | **2** | **0.0125** |
| Log mwj | CM | Right | **0.0075** | **3** | **0.0188** |
| FA | NCM | Right | **0.0121** | **4** | **0.0250** |
| Log mwj | CM | Left | **0.0126** | **5** | **0.0313** |
| FA | tFA | Left | 0.4200 | 6 | 0.0375 |
| FA | tFA | Right | 0.8940 | 7 | 0.0438 |
| FA | NCM | Left | 0.1060 | 8 | 0.0500 |
