# Supplementary material for: In vivo assessment of the neural substrate linked with vocal imitation accuracy
Source: eLife. 2020 Mar 20;9:e49941. doi: 10.7554/eLife.49941 (PMC7083600; doi:10.7554/eLife.49941)
Supplement: Supplementary file 7. — 'log mwj’ refers to the log-transformed, modulated and warped jacobian determinants; FA stands for Fractional Anisotropy, one of the DTI metrics. rmcorr’ is the repeated-measures correlation analysis. FDR rate = 0.05; number of tests = 16; i is the rank, m is the total number of tests and Q is the false discovery rate set at 0.05. Only those tests that survive FDR correction for multiple comparisons are highlighted in bold. [file elife-49941-supp7.docx]

**Supplementary file 7: Benjamini-Hochberg FDR correction for multiple comparisons of Spearmans’ ρ analyses.**

| **MRI parameter** | **Cluster-based ROI** | **Age** | **Hemisphere** | ***p* value** | **rank** | **(i/m)Q** |
| --- | --- | --- | --- | --- | --- | --- |
| FA | VP | 65 |  | **0.0004** | **1** | **0.0031** |
| Log mwj | VP | 65 |  | **0.0006** | **2** | **0.0063** |
| FA | VP | 200 |  | **0.0008** | **3** | **0.0094** |
| FA | tFA | 65 | Left | **0.0009** | **4** | **0.0125** |
| FA | tFA | 200 | Left | **0.0012** | **5** | **0.0156** |
| FA | tFA | 200 | Right | **0.0041** | **6** | **0.0188** |
| FA | tFA | 65 | Right | **0.0045** | **7** | **0.0219** |
| FA | NCM | 200 | Left | **0.0070** | **8** | **0.0250** |
| FA | NCM | 65 | Left | **0.0081** | **9** | **0.0281** |
| FA | NCM | 65 | Right | **0.0138** | **10** | **0.0313** |
| FA | NCM | 200 | Right | **0.0336** | **11** | **0.0344** |
| Log mwj | CM | 65 | Left | 0.0514 | 12 | 0.0375 |
| Log mwj | CM | 65 | Right | 0.0846 | 13 | 0.0406 |
| Log mwj | VP | 200 |  | 0.1138 | 14 | 0.0438 |
| Log mwj | CM | 200 | Left | 0.2882 | 15 | 0.0469 |
| Log mwj | CM | 200 | Right | 0.5126 | 16 | 0.0500 |
