# Supplementary material for: In vivo assessment of the neural substrate linked with vocal imitation accuracy
Source: eLife. 2020 Mar 20;9:e49941. doi: 10.7554/eLife.49941 (PMC7083600; doi:10.7554/eLife.49941)
Supplement: Supplementary file 8. — This table summarises the results of the mixed-effect model analyses testing for an interaction between age (20-30-40 dph) and future good-bad learning outcome (n = 14 birds). The mixed-effect model includes a fixed effect for age and for learning outcome (good-bad), and a random factor for bird identity. The restricted Maximum Likelihood method was used to fit the data and significance was assessed using F-tests with the Kenward-Roger approximation. [file elife-49941-supp8.docx]

**Supplementary file 8: Summary of mixed-effect model.**

| **MRI parameter** | **Cluster-based ROI** | **Hemisphere** | **Interaction good-bad*age** | **Main effect good-bad** |
| --- | --- | --- | --- | --- |
| **FA** | **NCM** | Left | *p*=0.1146 *F_(2,19.6)_*=2.4242 | *p*=0.0003 *F_(1,10.2)_*=28.3681 |
|  |  | Right | *p*=0.0289 *F_(2,19.4)_*=4.2797 | *p*=0.2550 *F_(1,10.1)_*=1.4557 |
|  | **tFA** | Left | *p*=0.1378 *F_(2,19.4)_*=2.1989 | *p*=0.0998 *F_(1,10.1)_*=3.2809 |
|  |  | Right | *p*=0.0113 *F_(2,19.2)_*=5.7186 | *p*=0.1198 *F_(1,10.1)_*=2.8898 |
|  | **VP** |  | *p*=0.5908 *F_(2,19.3)_*=0.5409 | *p*=0.5856 *F_(1,10.0)_*=0.3173 |
| **log mwj** | **VP** |  | *p*=0.6713 *F_(2,20.0)_*=0.4066 | *p*=0.0463 *F_(1,10.0)_*=5.1686 |
|  | **CM** | Left | *p*=0.1188 *F_(2,20.0)_*=2.3740 | *p*=0.3280 *F_(1,10.0)_*=1.0577 |
|  |  | Right | *p*=0.4105 *F_(2,20.0)_*=0.9313 | *p*=0.5240 *F_(1,10.0)_*=0.4359 |
