# Supplementary material for: In vivo assessment of the neural substrate linked with vocal imitation accuracy
Source: eLife. 2020 Mar 20;9:e49941. doi: 10.7554/eLife.49941 (PMC7083600; doi:10.7554/eLife.49941)
Supplement: Supplementary file 9. — FDR rate = 0.05; number of tests = 8; i is the rank, m is the total number of tests and Q is the false discovery rate set at 0.05; None of the tests survives FDR correction. [file elife-49941-supp9.docx]

**Supplementary file 9: Benjamini-Hochberg FDR correction for multiple comparisons of the interaction good-bad*age.**

| **MRI parameter** | **Cluster-based ROI** | **Hemisphere** | ***p* value** | **rank** | **(i/m)Q** |
| --- | --- | --- | --- | --- | --- |
| FA | tFA | Right | 0.0113 | 1 | 0.0063 |
| FA | NCM | Right | 0.0289 | 2 | 0.0125 |
| FA | NCM | Left | 0.1146 | 3 | 0.0188 |
| Log mwj | CM | Left | 0.1188 | 4 | 0.0250 |
| FA | tFA | Left | 0.1378 | 5 | 0.0313 |
| Log mwj | CM | Right | 0.4105 | 6 | 0.0375 |
| FA | VP |  | 0.5908 | 7 | 0.0438 |
| Log mwj | VP |  | 0.6713 | 8 | 0.0500 |
