# Supplementary material for: In vivo assessment of the neural substrate linked with vocal imitation accuracy
Source: eLife. 2020 Mar 20;9:e49941. doi: 10.7554/eLife.49941 (PMC7083600; doi:10.7554/eLife.49941)
Supplement: Supplementary file 11. — FDR rate = 0.05; number of tests = 5; i is the rank, m is the total number of tests and Q is the false discovery rate set at 0.05. Only those tests that survive FDR correction for multiple comparisons are highlighted bold. [file elife-49941-supp11.docx]

**Supplementary file 11: Benjamini-Hochberg FDR correction for multiple comparisons of the spearman’s correlation between % song similarity at 200 dph and FA at 20 dph.**

| **Cluster-based ROI** | **Hemisphere** | **Spearman’s ρ** | ***p* value** | **rank** | **(i/m)Q** |
| --- | --- | --- | --- | --- | --- |
| NCM | Left | 0.662 | **0.01** | **1** | **0.0100** |
| NCM | Right | 0.578 | 0.03 | 2 | 0.0200 |
| tFA | Right | 0.499 | 0.069 | 3 | 0.0300 |
| tFA | Left | 0.468 | 0.091 | 4 | 0.0400 |
| VP |  | 0.191 | 0.513 | 5 | 0.0500 |
