# Supplementary material for: In vivo assessment of the neural substrate linked with vocal imitation accuracy
Source: eLife. 2020 Mar 20;9:e49941. doi: 10.7554/eLife.49941 (PMC7083600; doi:10.7554/eLife.49941)
Supplement: Supplementary file 12. [file elife-49941-supp12.docx]

**Supplementary file 10: Detailed information on bio-parents, foster-parents, pupils and tutors.**
